# Phosphate reabsorption in the kidney: NaPi-IIb or not IIb

**DOI:** 10.1007/s00424-020-02374-5

**Published:** 2020-04-01

**Authors:** David Burns, Andreas Werner

**Affiliations:** Biosciences Institute, Newcastle University, Framlington Place, Newcastle upon Tyne, NE2 4HH UK

Phosphate is an essential component of nucleic acids, cell membranes and bones, and is important for enzymatic interactions, synthesis of ATP and other signalling and metabolic pathways. The level of inorganic phosphate (Pi) in serum is tightly regulated involving intestinal uptake of dietary Pi, storage in bone and excretion of excess Pi in the kidney. Even slight disruptions in the balance of serum Pi can lead to major disorders ranging from cardiovascular and kidney disease if serum phosphate is too high to bone demineralisation if serum Pi is too low [5]. Two protein families of Na-dependent Pi transporters, SLC34A1, SLC34A2 and SLC34A3 (NaPi-IIa, NaPi-IIb, NaPi-IIc) as well as SLC20A1 and SLC20A2 (Pit-1 and Pit-2) are central to maintaining Pi homeostasis [2]. Since the molecular identification of these transporters, SLC34A1 and SLC34A3 have emerged as the ‘renal’ isoforms, SLC34A2 as the ‘intestinal’ isoform and SLC20A1/2 as widely expressed ‘housekeeping’ transporters [2].

**Fig. 1:**
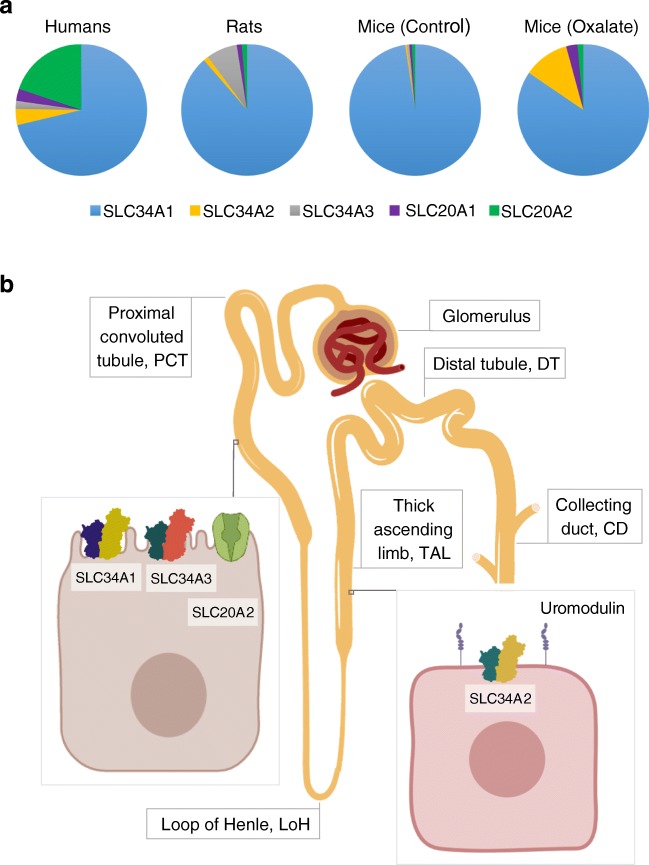
Pi reabsorption in the kidney of humans, rats and mice. **a** Data adapted from Motta and Silva et al. illustrates that SLC34A1 is by far the most abundant sodium phosphate transporter in the kidneys of all three species examined. In mice with oxalate-induced kidney damage, the proportion of Slc34a2 increases considerably. **b** SLC34A1, SLC34A3 and SLC20A2 are expressed in cells in the proximal tubule (PCT), whereas SLC34A2 co-localizes with uromodulin in cells of the thick ascending loop of Henle (TAL)

The main mechanism of regulating serum Pi is via modifying the reuptake of Pi in the proximal tubule of kidneys. SLC34A1 (NaPi-IIa) is the main transporter involved in Pi reuptake, SLC34A3 (NaPi-IIc) plays an essential role during growth whereas SLC20A2 (PiT-2) contributes to a lesser extent [7]. Pi reabsorption in the distal parts of the renal tubule, ‘fine-tuning Pi excretion in the urine’ has long been established, though the molecular mediators of distal Pi uptake have not been identified [1]. The findings from Carsten Wagner’s lab published in this issue demonstrating the expression of SLC34A2 in distal parts of the renal tubule appear to close this knowledge gap (Fig. 1).

In their comprehensive study, Sarah E. Motta and Pedro Henrique Imenez Silva et al. compare the expression of sodium phosphate transporters in kidneys of humans, mice and rats. Not surprisingly, they find that the most abundant transporter in all three species was SLC34A1. SLC34A1 and SLC34A3 were expressed in overlapping areas in the proximal tubule, whereas SLC34A2 appears to co-localize with uromodulin in the thick ascending loop of Henle and does not overlap with the expression of SLC34A1 or SLC34A3.

Motta and Silva et al. also demonstrate that mice fed a high Pi diet did not appear to have altered Pi transporter mRNA levels compared with those fed a low Pi diet. They suggest that adaptive changes in response to dietary Pi may occur at the protein level, rather than at a transcriptional level in agreement with previous reports [6]. Moreover, mice lacking *Slc34a2* and *Slc34a3* did not show compensatory mRNA upregulation of other Pi transporters.

Finally, when kidney damage was induced in mice by administering a high oxalate diet, a common murine model for chronic kidney disease [3, 4], Motta and Silva et al. show that whilst *Slc34a1* and *Slc34a3* are downregulated, these mice upregulate *Slc34a2*. This finding may have interesting implications in managing hyperphosphataemia in patients with chronic kidney disease. A first step will be to assess whether patients with kidney disease show an upregulation in SLC34A2. Moreover, it will be interesting to see how drugs that increase urinary Pi excretion and primarily act in the distal tubule such as diuretics affect intracellular location or transport functions of SLC34A2.

To conclude, these findings are an important contribution to the overall knowledge of Pi homeostasis, particularly with regard to the expression of SLC34A2 in the kidney and the comparative mapping of the different Pi transporters. It would be commendable to expand similar studies to include groups of various ages throughout the entire lifespan to monitor growth-related expression changes. Such information is crucial to evaluate the complex phenotypes presented in patients with mutated Pi transporters and to understand how our body strikes the fine balance between too little Pi at young age and too much Pi during adulthood.
